# MDSC-derived S100A8/9 contributes to lupus pathogenesis by promoting TLR7-mediated activation of macrophages and dendritic cells

**DOI:** 10.1007/s00018-024-05155-w

**Published:** 2024-03-01

**Authors:** Yonghong Yang, Xin Zhang, Lina Jing, Yucai Xiao, Yangzhe Gao, Yuxin Hu, Shujiao Jia, Guangxi Zhou, Huabao Xiong, Guanjun Dong

**Affiliations:** 1 Medical Research Center, Affiliated Hospital of Jining Medical University, Jining, 272029 Shandong China; 2 Institute of Immunology and Molecular Medicine, Jining Medical University, Jining, 272067 Shandong China; 3 Jining Key Laboratory of Immunology, Jining Medical University, Jining, 272067 Shandong China

**Keywords:** SLE, MDSCs, Macrophage, TLR7, S100A8/9

## Abstract

Toll-like receptors (TLRs), especially TLR7, play an important role in systemic lupus erythematosus (SLE) pathogenesis. However, the regulatory mechanism underlying the abnormal activation of TLR pathways in patients with SLE has not been elucidated. Notably, accumulating evidence indicates that myeloid-derived suppressor cells (MDSCs) are important regulators of inflammation and autoimmune diseases. Compared with healthy control subjects, patients with SLE have a greater proportion of MDSCs among peripheral blood mononuclear cells (PBMCs); however, the effect of MDSCs on TLR7 pathway activation has not been determined. In the present study, lupus MDSCs significantly promoted TLR7 pathway activation in macrophages and dendritic cells (DCs), exacerbating the imiquimod-induced lupus model. RNA-sequencing analysis revealed significant overexpression of S100 calcium-binding protein A8 (S100A8) and S100A9 in MDSCs from diseased MRL/*lpr* mice. In vitro and in vivo studies demonstrated that S100A8/9 effectively promoted TLR7 pathway activation and that S100A8/9 deficiency reversed the promoting effect of MDSCs on TLR7 pathway activation in lupus. Mechanistically, MDSC-derived S100A8/9 upregulated interferon gamma (IFN-γ) secretion by macrophages and IFN-γ subsequently promoted TLR7 pathway activation in an autocrine manner. Taken together, these findings suggest that lupus MDSCs promote TLR7 pathway activation and lupus pathogenesis through the S100A8/9-IFN-γ axis. Our study identified an important target for SLE therapy.

**Supplementary Information:**

The online version contains supplementary material available at 10.1007/s00018-024-05155-w.

## Introduction

Systemic lupus erythematosus (SLE) is a complex, heterogeneous, systemic autoimmune disease associated with multiorgan damage [1]. While SLE pathogenesis involves various factors, abnormal activation of the immune system has been identified as the core factor for pathogenesis [2]. Deregulated activation of immune cells, such as monocytes/macrophages, dendritic cells (DCs), and B cells, can lead to the breakdown of self-tolerance and is associated with SLE pathogenesis [3–5]. For example, activated monocytes/macrophages infiltrate target organs and secrete numerous proinflammatory factors [3]; activated plasmacytoid DCs (pDCs) induce excessive production of type I interferon (IFN), which directly primes autoreactive B and T cells and mediates DC-induced autoreactive T-cell activation [4]; and activated myeloid DCs (mDCs) produce massive amounts of proinflammatory cytokines and aberrantly present autoantigens, driving the autoreactivity of T and B cells [5]. Therefore, elucidating the mechanism underlying the abnormal activation of immune cells is helpful for elucidating SLE pathogenesis and providing new ideas for the clinical treatment of SLE.

Toll-like receptors (TLRs) are evolutionarily conserved pattern recognition receptors that are critical for mediating innate and adaptive immunity by detecting potentially harmful pathogens [6, 7]. TLR7 exclusively recognizes single-stranded RNA and activates type I IFN signaling and nuclear factor kappa-light-chain enhancer of activated B cells (NF-κB)-inducible pathways, leading to the differentiation and activation of immune cells, such as monocytes/macrophages, DCs, and B cells [8, 9]. It has been recognized that TLR7 plays an important role in SLE pathogenesis. Genetic studies on SLE in humans revealed the TLR7 rs3853839 C/G single-gene polymorphism [10, 11]. Excessive activation of the TLR7 pathway can aggravate lupus pathogenesis by driving the expansion of B cells and autoantibody production [12, 13]. TLR7 deficiency effectively alleviates the symptoms of lupus mice, while TLR7 overexpression triggers lupus-like autoimmune disease [14, 15]. Despite extensive research on how abnormal expression of TLR7 contributes to SLE development, the cellular mechanisms driving TLR7 activation have not been fully elucidated.

Myeloid-derived suppressor cells (MDSCs) are a heterogeneous group of immature myeloid cells that include early myeloid progenitors, immature macrophages, granulocytes, and DCs [16]. Under pathological conditions such as inflammation or tumorigenesis, the normal differentiation of immature bone marrow cells is inhibited, leading to the expansion and activation of MDSCs with immunomodulatory activity [17]. MDSCs can be broadly characterized as HLA-DR^−^CD11b^+^CD14^−^CD33^+^ in humans and as CD11b^+^Gr-1^+^ in mice [18]. Recently, the role of MDSCs in various autoimmune diseases, including SLE, has garnered significant attention. Although MDSCs exhibit significant expansion in patients with SLE and in a lupus mouse model and the cell proportion is positively correlated with disease severity [19, 20], the exact function of MDSCs in SLE pathogenesis remains controversial. Several studies suggest that MDSCs play an immunosuppressive role in lupus pathogenesis by directly inhibiting the differentiation of naive B cells into antibody-secreting cells, suppressing autoreactive B and T cells, and inhibiting the activation of macrophages [21, 22], indicating that MDSCs could be a potential therapeutic option for SLE. Conversely, other studies propose that MDSCs do not have an immunosuppressive function in lupus but instead exert an immune-promoting function, particularly a pathogenic effect, by regulating the balance of the T helper 17 (Th17) cells and the regulatory T (Treg) cells [23–25]. However, the effect of MDSCs on TLR7-mediated autoimmune responses and lupus pathogensis has not been determined.

The calcium-binding proteins S100A8 and S100A9 belong to the S100 family and are mainly expressed by activated myeloid cells, including polymorphonuclear neutrophils, monocytes, and DCs. When S100A8/9 binds to its receptor, it induces the production of various cytokines, chemokines, and adhesion factors, thus participating in immune responses [26]. The levels of heterodimers of S100A8 and S100A9 (S100A8/9) are often elevated during inflammation and their immunostimulatory or immunoprotective properties are dependent on the underlying pathology [27]. Clinical data suggest that, compared with those in patients with inactive SLE and healthy individuals, the expression of S100A8 and S100A9 in patients with active SLE is significantly elevated and positively correlated with systemic lupus erythematosus disease activity index (SLEDAI) scores [28, 29]. Interestingly, a previous study showed that S100A9-deficient male NZBWF1 mice developed accelerated autoimmunity, while female mice showed neither a response to S100A9 deficiency nor even a slight reduction in disease symptoms [30]. However, whether S100A8/9 regulates TLR7 pathway activation and the TLR7-mediated autoimmune has not been determined.

In the present study, we investigated the role of MDSCs in TLR7-mediated lupus pathogenesis. Our results indicate that MDSCs may aggravate the pathogenesis of TLR7-mediated lupus by promoting the activation of macrophages and DCs through the S100A8/9-IFN-γ axis, suggesting that MDSC-derived S100A8/9 plays a crucial role in lupus pathogenesis. This study offers important insights into potential SLE therapies by targeting MDSCs.

## Materials and methods

### Mice

Female C57BL/6 mice were purchased from Pengyue Experimental Animal Breeding Company (Jinan, China), MRL-fas^lpr^ (MRL/*lpr*) mice were obtained from Aniphe Biolaboratory Inc. (Nanjing, China), and S100A9^−/−^ mice were purchased from Cyagen Biosciences Inc. (Guangzhou, China). The mice were housed in specific pathogen-free conditions at Jining Medical University. When grown to 10 weeks, the C57BL/6 or S100A9^−/−^ mice had 1.25 mg of 5% imiquimod cream (IMQ, Sichuan MED-SHINE Pharmaceutical Co., Ltd.) applied to the ear, three times per week. After 10 weeks treatment, mice were humanely killed and spleens, mesenteric lymph nodes (mLNs), kidneys and blood were collected for further study. All experiments were conducted in accordance with institutional guidelines for animal care and used in accordance with the Guide for the Animal Care Committee of Jining Medical University Affiliated Hospital.

### Antibodies

The following antibodies were used for immunoblotting: anti-p38, anti-p-p38, anti-Erk, anti-p-Erk, anti-JNK, anti-p-JNK, anti-p65, and anti-p-p65. The antibodies were purchased from Cell Signaling Technology and used at a 1:1000 dilution; anti-β-actin (diluted at 1:1000), HRP-labeled goat anti-rabbit (diluted at 1:3000) and HRP-labeled rabbit anti-mouse (diluted at 1:3000) were purchased from Beyotime Institute of Biotechnology. The following antibodies were used for flow cytometry: FITC-labeled anti-mouse B220, CD4 or F4/80, PE-labeled anti-mouse GL7, CD3e or CD40, APC-labeled anti-mouse CD95, CD138 or CD86, APC/Cy7-labeled anti-mouse CD11b, and BV421-labeled anti-mouse CD69 or CD11c. The antibodies were purchased from Biolegend and used at a 1:100 dilution. An isotype control was used for each antibody. The following antibodies were used for confocal immunofluorescence microscopy: Alexa Fluor 488-conjugated goat anti-mouse IgG (Invitrogen), Alexa Fluor 488-conjugated goat anti-mouse IgM (1:200, Invitrogen), anti-mouse S100A8 antibody (10 μg/mL, R&D) and anti-mouse S100A9 antibody (1:100, Invitrogen).

### Preparation of bone marrow-derived macrophages (BMDMs) and dendritic cells (BMDCs)

Bone marrow-derived macrophages (BMDMs) and dendritic cells (BMDCs) were obtained as previously described. In brief, bone marrow cells from tibias and femurs of C57BL/6 mice were collected and differentiated for 7 days in complete Dulbecco’s modified Eagle medium (DMEM, Gibco) containing granulocyte–macrophage colony stimulating factor (GM-SCF, 10 ng/mL, PeproTech) for BMDMs or RPMI 1640 medium (Gibco) containing GM-CSF (20 ng/mL) and IL-4 (1 ng/mL, PeproTech) for BMDCs, respectively.

### Isolation and adoptive transfer of MDSCs

Splenic MDSCs were isolated using a mouse MDSC isolation kit (Miltenyi Biotec) following the manufacturer’s protocol. For adoptive transfer experiment, MDSCs isolated from 22- to 24-week-old C57BL/6 and MRL/*lpr* mice were injected into 8-week-old C57BL/6 mice (2 × 10^6^ cells/mouse), once every 2 weeks for 10 weeks.

### Co-culture assay

Co-culture assay of BMDMs or BMDCs with MDSCs was performed using a transwell system. Briefly, splenic MDSCs (1 × 10^6^ cells), placed in transwell chambers, were co-cultured with BMDMs or BMDCs in 12-well plate at a 1:1 ratio. After 12 h, MDSCs were removed and BMDMs or BMDCs were stimulated with R848 (1 μg/mL, GLPbio) to detect the activation of the TLR7 pathway.

### Flow cytometry

For flow cytometry analysis, single cell suspensions were incubated with flow antibodies for 30 min at 4 ℃. After washing, cells were resuspended in PBS for flow cytometry. Data were acquired on a FACS Verse (Becton Dickinson) and analyzed by FlowJo software.

### RNA sequencing analysis

The splenic MDSCs, isolated from 6-week and 24-week-old MRL/*lpr* mice were sorted by magnetic beads and sent to Beijing Genomics Institute for RNA sequencing analysis. Briefly, total RNA was extracted and qualified and cDNA synthesized using random N6 primer. The sequencing was performed using BGISEQ-500.

### Immunofluorescence analysis

Mice kidneys were collected, paraffin embedded and sectioned as described previously. Kidney sections were blocked for 1 h with 3% bovine serum albumin (BSA). For immunofluorescence of IgG and IgM, kidney sections were stained overnight at 4 ℃ with Alexa Fluor 488-conjugated goat anti-mouse IgG or Alexa Fluor 488-conjugated goat anti-mouse IgM. After washing three times, the sections were mounted. For immunofluorescence of S100A8 and S100A9, kidney sections were stained overnight at 4 ℃ with anti-mouse S100A8 or anti-mouse S100A9 antibody. After washing three times, the sections were stained overnight at room temperature with FITC-rabbit anti-goat IgG or FITC-goat anti-rabbit IgG secondary antibody. After washing three times, the sections were mounted. Images were acquired using Zeiss LSM 800 confocal microscope.

### Enzyme-linked immunosorbent assay (ELISA)

The concentrations of IL-12/IL-23 p40 and TNF-α were measured using ELISA kits (Biolegend). ELISA detection was performed according to the manufacturer’s instructions. The data was read by ELx800 Absorbance Microplate Reader (BioTek, VT, USA) at 450 nm with 630 nm as the correction wavelength.

### Quantitative real-time PCR (Q-PCR)

Total RNA was extracted using TRIzol reagent (Takara) and reverse transcribed to cDNA according to the manufacturer’s instructions. Real-time PCR assay was performed as described previously. Gene expression was normalized to GAPDH and analyzed with standard 2^−ΔΔCt^ method.

### Immunoblotting analysis

Total protein extraction was obtained as described previously. Protein samples were separated and transferred to PVDF membranes (Millipore). After blocking with 3% BSA, membranes were incubated with primary antibodies overnight at 4 °C, followed by incubation with HRP-conjugated secondary antibodies. Protein bands were visualized using ECL plus western blotting detection reagents (Thermo Fisher Scientific). β-Actin was used as an internal control.

### Data analysis

Statistical analyses were performed using Prism software (GraphPad). Significance was determined by Student’s *t* test or one-way ANOVA. A statistical significance level was set at *p* < 0.05.

## Results

### Lupus MDSCs aggravate disease progression in the TLR7 agonist IMQ-induced lupus mice

**Fig. 1:**
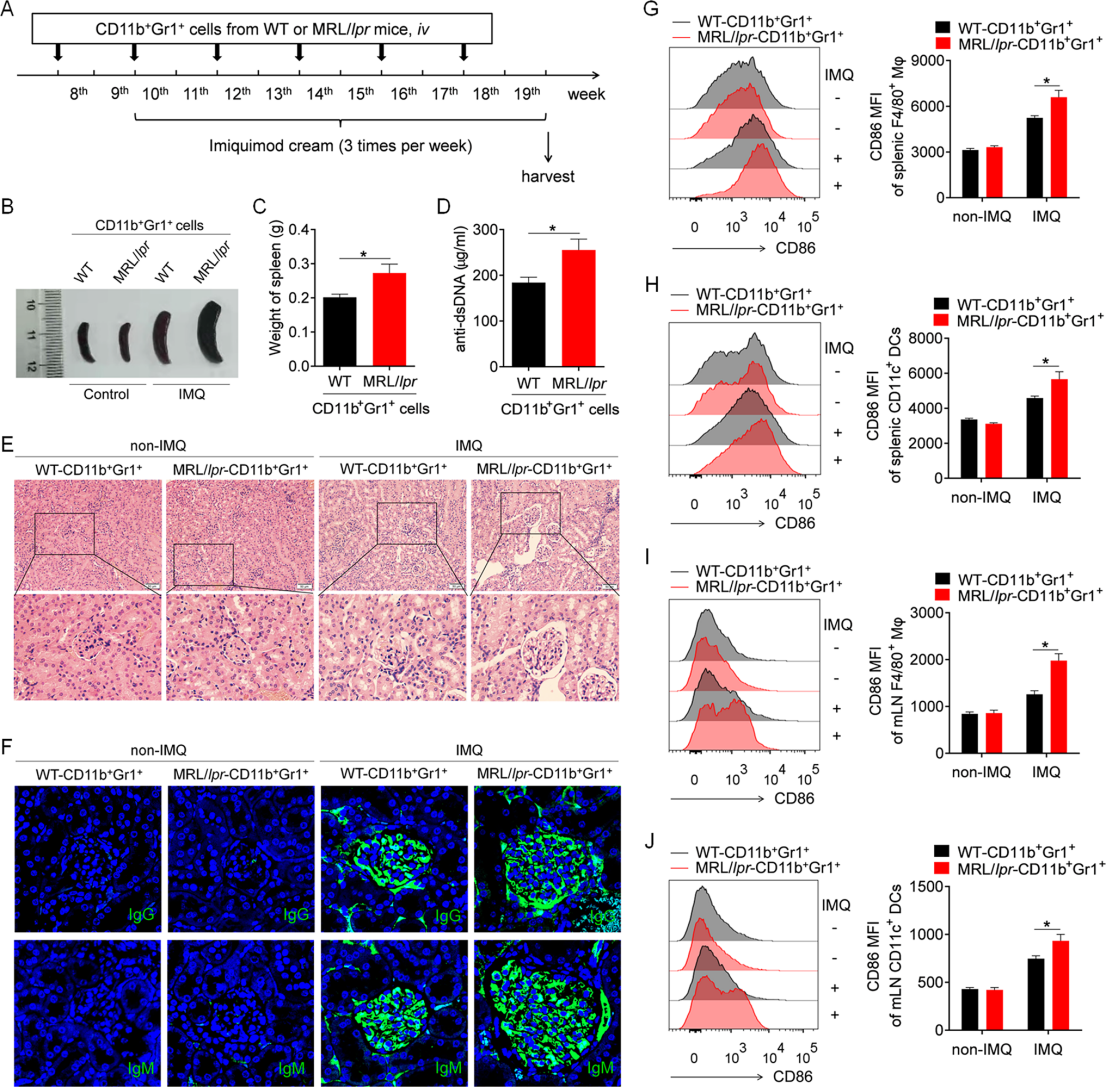
Lupus MDSCs contribute to the disease progression of mice with lupus induced by imiquimide (IMQ). The splenic MDSCs isolated from 22- to 24-week-old C57BL/6 mice or MRL/*lpr* mice were transferred into 8-week-old C57BL/6 mice by caudal vein (*n* = 6), 2 × 10.^6^ cells/mice, once every 2 weeks. At the 10th week, mice were treated with IMQ to induce lupus model. After 10 weeks, the mice were killed for further study. **A** The schematic showing the experimental design. **B** Representative images of marked splenomegaly. **C** Weight of spleens. **D** ELISA analysis of the anti-dsDNA antibody in serum. **E** Representative images of kidney sections detected by H&E. **F** Immunofluorescence of IgG and IgM of kidney sections. **G**–**J** Flow cytometric analysis of the expression of CD86 on macrophages and dendritic cells in spleens (**G**, **H**) and mLN (**I**, **J**) of mice. *p < 0.05

To study the effect of MDSCs on the pathogenesis of imiquimod (IMQ)-induced lupus in mice, we first investigated the effect of adoptive transfer of lupus MDSCs isolated from diseased MRL/*lpr* mice on disease progression in IMQ-induced lupus mice. Briefly, splenic MDSCs isolated from C57BL/6 mice (control MDSCs) and diseased MRL/*lpr* mice (lupus MDSCs) were adoptively transferred to IMQ-treated C57BL/6 mice (Fig. 1A). Intriguingly, compared with control MDSCs, lupus MDSCs significantly promoted disease progression in IMQ-treated mice. The disease mainly manifested as marked splenomegaly (Fig. 1B, C), increased levels of anti-double-stranded DNA (dsDNA) antibody (Fig. 1D), severe infiltration of lymphoid cells and diffuse expansion of the mesangial matrix in kidneys (Fig. 1E), and increased glomerular deposition of IgG and IgM antibodies (Fig. 1F).

To further investigate the effect of lupus MDSCs on the IMQ-induced lupus mouse model, we analyzed the activation of macrophages and DCs in the spleens of IMQ-induced lupus mice. As expected, compared with IMQ-treated mice that received control MDSCs, IMQ-treated mice that received lupus MDSCs showed significantly higher levels of CD86 on splenic macrophages (Fig. 1G) and DCs (Fig. 1H). Moreover, compared with control MDSCs, lupus MDSCs significantly promoted the activation of splenic B cells (Supplementary Fig. 1A, B) and CD4^+^ T cells (Supplementary Fig. 1C, D), and increased the percentage of germinal center B cells (Supplementary Fig. 1E, F) and plasmacytes (Supplementary Fig. 1G, H) in the spleen.

Furthermore, the aforementioned results were confirmed in the lymph nodes. As expected, a similar phenomenon was observed in the lymph nodes; that is, compared with control MDSCs, lupus MDSCs significantly promoted the activation of macrophages (Fig. 1I), DCs (Fig. 1J), B cells (Supplementary Fig. 2A, B), and CD4^+^ T cells (Supplementary Fig. 2C, D) and increased the percentage of germinal center B cells (Supplementary Fig. 2E, F) and plasmacytes (Supplementary Fig. 2G, H) in mesenteric lymph nodes (mLNs). In summary, lupus MDSCs can significantly promote abnormal activation of the immune system and aggravate disease progression in mice with lupus induced by the TLR7 agonist IMQ.

### Lupus MDSCs promote TLR7-mediated activation of macrophages and DCs in vitro

**Fig. 2:**
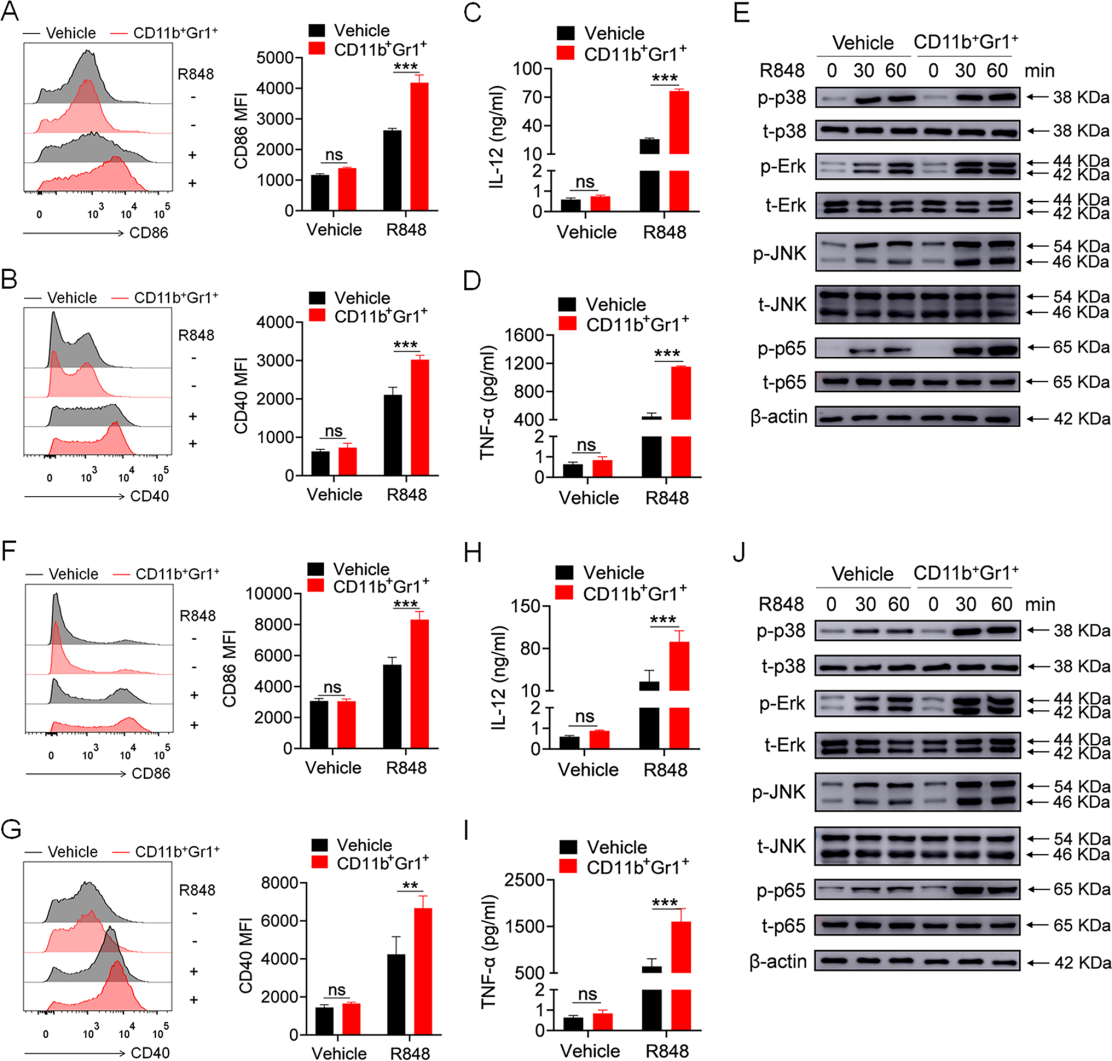
Lupus MDSCs promote the TLR7-mediated activation of BMDMs and BMDCs in vitro. Splenic MDSCs, isolated from 22 to 24 weeks MRL/*lpr* mice, were placed in transwell chambers and co-cultured with BMDMs or BMDCs at a 1:1 ratio (1 × 10^6^ cells). Twelve hours later, MDSCs were removed and R848 (1 μg/mL) was added into the culture system. **A**, **B** Flow cytometric analysis of the expression of CD86 and CD40 on BMDMs at 24 h. **C**, **D** ELISA analysis of the levels of IL-12/IL-23 p40 and TNF-α secreted by BMDMs at 24 h. **E** Western blot analysis of the phosphorylation of p38, Erk, JNK, and p65 in BMDMs at 30 and 60 min. **F**, **G** Flow cytometric analysis of the expression of CD86 and CD40 on BMDCs at 24 h. **H**, **I** ELISA analysis of the levels of IL-12/IL-23 p40 and TNF-α secreted by BMDCs at 24 h. **J** Western blot analysis of the phosphorylation of p38, Erk, JNK and p65 in BMDCs at 30 and 60 min. **p < 0.01, ***p < 0.001

We then explored the regulatory effect of lupus MDSCs on the activation of bone marrow-derived macrophages (BMDMs) and dendritic cells (BMDCs) induced by the TLR7 agonist R848. Splenic MDSCs isolated from MRL/*lpr* mice were co-cultured with BMDMs or BMDCs at a 1:1 ratio. After 12 h, the MDSCs were removed, and R848 was added to the culture system. Lupus MDSCs significantly strengthened the R848-induced expression of CD86 and CD40 in BMDMs (Fig. 2A, B) as well as the secretion of interleukin (IL)-12 and tumor necrosis factor (TNF)-α by BMDMs (Fig. 2C, D). TLR7 mediates immune cell activation by activating the mitogen-activated protein kinase (MAPK) and NF-κB pathways. Thus, we explored the activation of the MAPK and NF-κB pathways and discovered that lupus MDSCs significantly enhanced the R848-induced phosphorylation of p38, Erk, JNK, and p65 in BMDMs (Fig. 2E).

We also observed the same phenomenon in BMDCs. Lupus MDSCs significantly strengthened the R848-induced expression of CD86 and CD40 in BMDCs (Fig. 2F, G), the secretion of IL-12 and TNF-α by BMDCs (Fig. 2H, I), and the phosphorylation of p38, Erk, JNK, and p65 in BMDCs (Fig. 2J). Collectively, these data indicated that lupus MDSCs could promote the TLR7-mediated activation of macrophages and DCs in vitro.

### S100A8/9 is highly expressed in MDSCs from diseased MRL/*lpr* mice

**Fig. 3:**
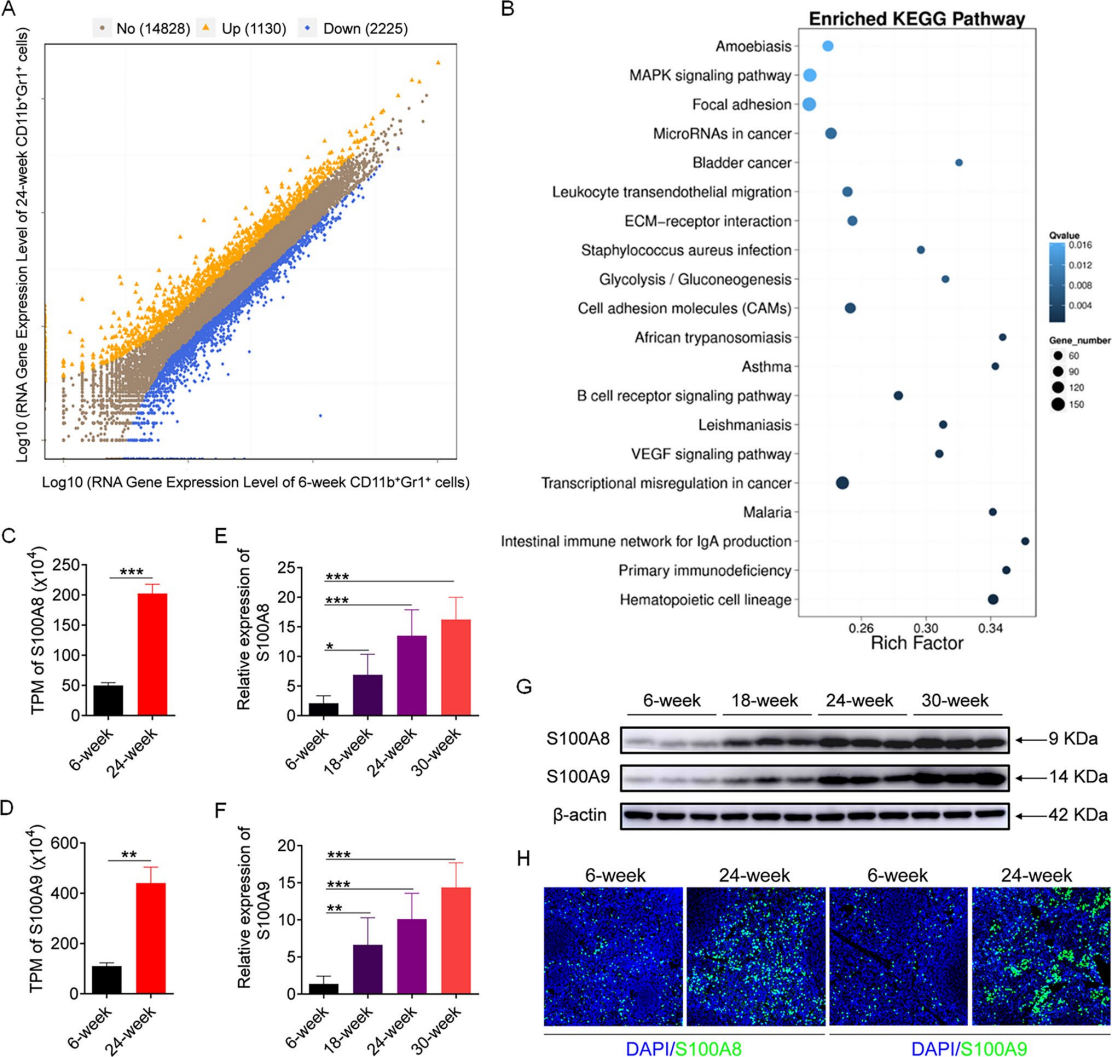
S100A8/9 is overexpressed in lupus MDSCs. (A-D) Splenic MDSCs were isolated from 6-week-old and 24-week-old MRL/*lpr* mice and then RNA-seq analysis was performed. **A** Differential expression genes at FDR < 0.05 in MDSCs from 24-week-old MRL/*lpr* mice versus those from 6-week-old MRL/*lpr* mice. **B** KEGG signaling pathway enrichment analysis of differential gene expression in MDSCs from 24-week-old MRL/*lpr* mice versus those from 6-week-old MRL/*lpr* mice. **C**, **D** TPM values of S100A8 and S100A9 analyzed by RNA-seq. **E**–**G** Splenic MDSCs were isolated from MRL/*lpr* mice of different ages (6, 12, 18 and 24 week), and the expression of S100A8 and S100A9 were analyzed by Q-PCR (**E**, **F**) and western blot (**G**). **H** Immunofluorescence staining of S100A8 and S100A9 in spleens of 6-week-old and 24-week-old MRL/*lpr* mice. *p < 0.05, **p < 0.01, ***p < 0.001

To determine how lupus MDSCs strengthen TLR7 pathway activation, we performed RNA-seq analysis of splenic MDSCs from 6-week-old and 24-week-old MRL/*lpr* mice. Overall, 1130 genes exhibited upregulated expression, and 2225 genes exhibited downregulated expression in MDSCs from 24-week-old MRL/*lpr* mice compared with 6-week-old MRL/*lpr* mice (Fig. 3A). Kyoto Encyclopedia of Genes and Genomes (KEGG) signaling pathway enrichment analysis revealed that the differentially expressed genes (DEGs) were related to multiple signaling pathways, such as those related to hematopoietic cell lineage and primary immunodeficiency and so on (Fig. 3B). GO analysis based on biological process (BP) revealed 30 enriched BP pathways from DEGs with an FDR, GO analysis based on cellular component (CC) revealed 19 enriched CC pathways from DEGs with an FDR, and GO analysis based on molecular function (MF) revealed 14 enriched MF pathways from DEGs with an FDR (Supplementary Fig. 3).

Among the top differentially expressed genes, S100A8 and S100A9 attracted our attention, because they had large differential expression ratios. Additionally, recent studies have confirmed that MDSCs participate in immune regulation by secreting S100A8/9. Notably, compared with those in MDSCs from 6-week-old MRL/*lpr* mice, S100A8 and S100A9 expression in MDSCs from 24-week-old MRL/*lpr* mice was nearly fourfold greater (Fig. 3C, D). To further verify the changes in S100A8/9 expression in lupus MDSCs, splenic MDSCs were isolated from MRL/*lpr* mice of different ages, and S100A8 and S100A9 expression was detected. As expected, S100A8 and S100A9 expression was upregulated with increasing age at the mRNA and protein levels (Fig. 3E–G). Moreover, the results of the immunofluorescence assay showed that S100A8 and S100A9 expression in the spleens of 24-week-old MRL/*lpr* mice was significantly greater than that in the spleens of 6-week-old MRL/*lpr* mice (Fig. 3H). Taken together, these results suggest that S100A8/9 is highly expressed in MDSCs from diseased MRL/*lpr* mice.

### Lupus MDSCs contribute to the activation of the TLR7 pathway through a S100A8/9-dependent mechanism

**Fig. 4:**
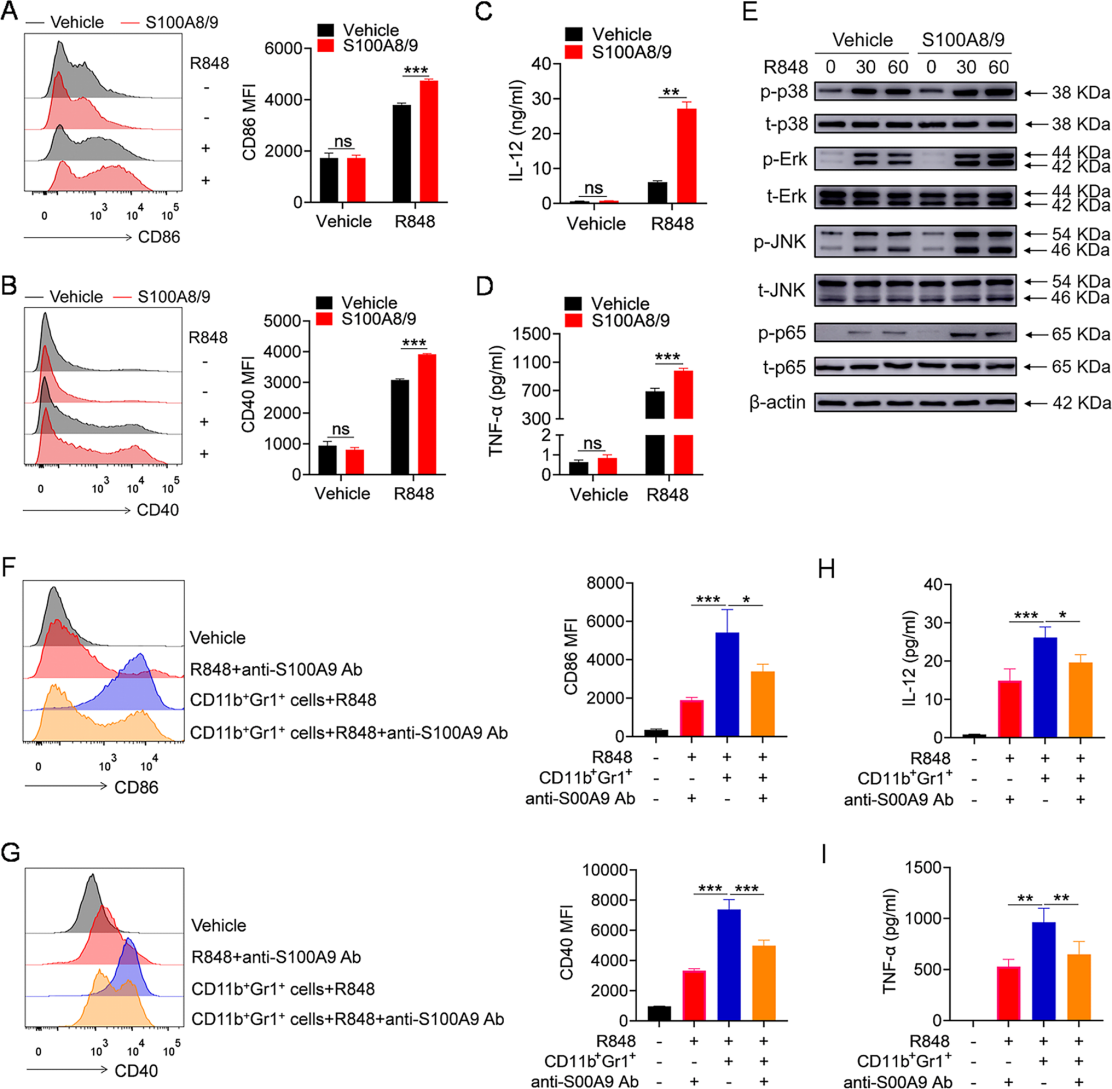
Lupus MDSCs promote the TLR7-mediated activation of BMDMs by secreting S100A8/9. **A–E** BMDMs were pretreated with mouse recombinant S100A8/9 protein (1 μg/mL) for 12 h, and then stimulated with R848 (1 μg/mL). Flow cytometric analysis of the expression of CD86 and CD40 at 24 h (**A**, **B**). ELISA analysis of the levels of IL-12/IL-23 p40 and TNF-α in culture supernatant at 24 h (**C**, **D**). Western blot analysis of the phosphorylation of p38, Erk, JNK and p65 at 30 and 60 min (**E**). **F**–**I** Splenic MDSCs, isolated from 22 to 24-week-old MRL/*lpr* mice, were placed in transwell chambers and co-cultured with BMDMs or BMDCs at a 1:1 ratio (1 × 10^6^ cells). Meanwhile, anti-S100A9 antibody or vehicle (1 μg/mL) was added. After 12 h, MDSCs were removed and R848 (1 μg/mL) was added into the culture system. Flow cytometric analysis of the expression of CD86 and CD40 at 24 h (**F**, **G**). ELISA analysis of the levels of IL-12/IL-23 p40 and TNF-α in culture supernatant at 24 h (**H**, **I**). *p < 0.05, **p < 0.01, ***p < 0.001

To explore whether S100A8/9 contributes to the promotion effect of lupus MDSCs through activation of the TLR7 pathway, we first investigated whether S100A8/9 can regulate the TLR7 pathway activation. Pretreatment with the mouse recombinant S100A8/9 protein significantly promoted R848-induced expression of CD86 and CD40 in BMDMs (Fig. 4A, B) as well as the secretion of IL-12 and TNF-α by BMDMs (Fig. 4C, D). Moreover, S100A8/9 promoted the activation of the MAPKs and NF-κB pathways in BMDMs, as the R848-induced phosphorylation of the p38, Erk, JNK, and p65 proteins was enhanced by S100A8/9 (Fig. 4E). Consistent with these findings, similar results were obtained for BMDCs. S100A8/9 significantly promoted R848-induced expression of CD86 and CD40 in BMDCs (Supplementary Fig. 4A, B); secretion of IL-12 and TNF-α by BMDCs (Supplementary Fig. 4C, D); and phosphorylation levels of p38, Erk, JNK, and p65 in BMDCs (Supplementary Fig. 4E). These data suggest that S100A8/9 indeed promotes TLR7 pathway activation.

To confirm that MDSCs promote TLR7 pathway activation by secreting S100A8/9, splenic MDSCs isolated from 24-week-old MRL/*lpr* mice were co-cultured with BMDMs, and an anti-S100A9 neutralizing antibody or vehicle was added. After 12 h, the MDSCs were removed, and R848 was added to the culture medium. Intriguingly, lupus MDSCs significantly promoted R848-induced expression of CD86 and CD40 on BMDMs and the secretion of IL-12 and TNF-α by BMDMs, whereas neutralizing S100A9 markedly reversed the promoting effect of lupus MDSCs on R848-induced expression of CD86 and CD40 on BMDMs (Fig. 4F, G) and the secretion of IL-12 and TNF-α by BMDMs (Fig. 4H, I). These results suggest that, to some extent, lupus MDSCs promote TLR7 pathway activation in a manner dependent on S100A8/9.

### S100A8/9 contributes to TLR7 pathway activation in BMDMs by promoting the autosecretion of IFN-γ

**Fig. 5:**
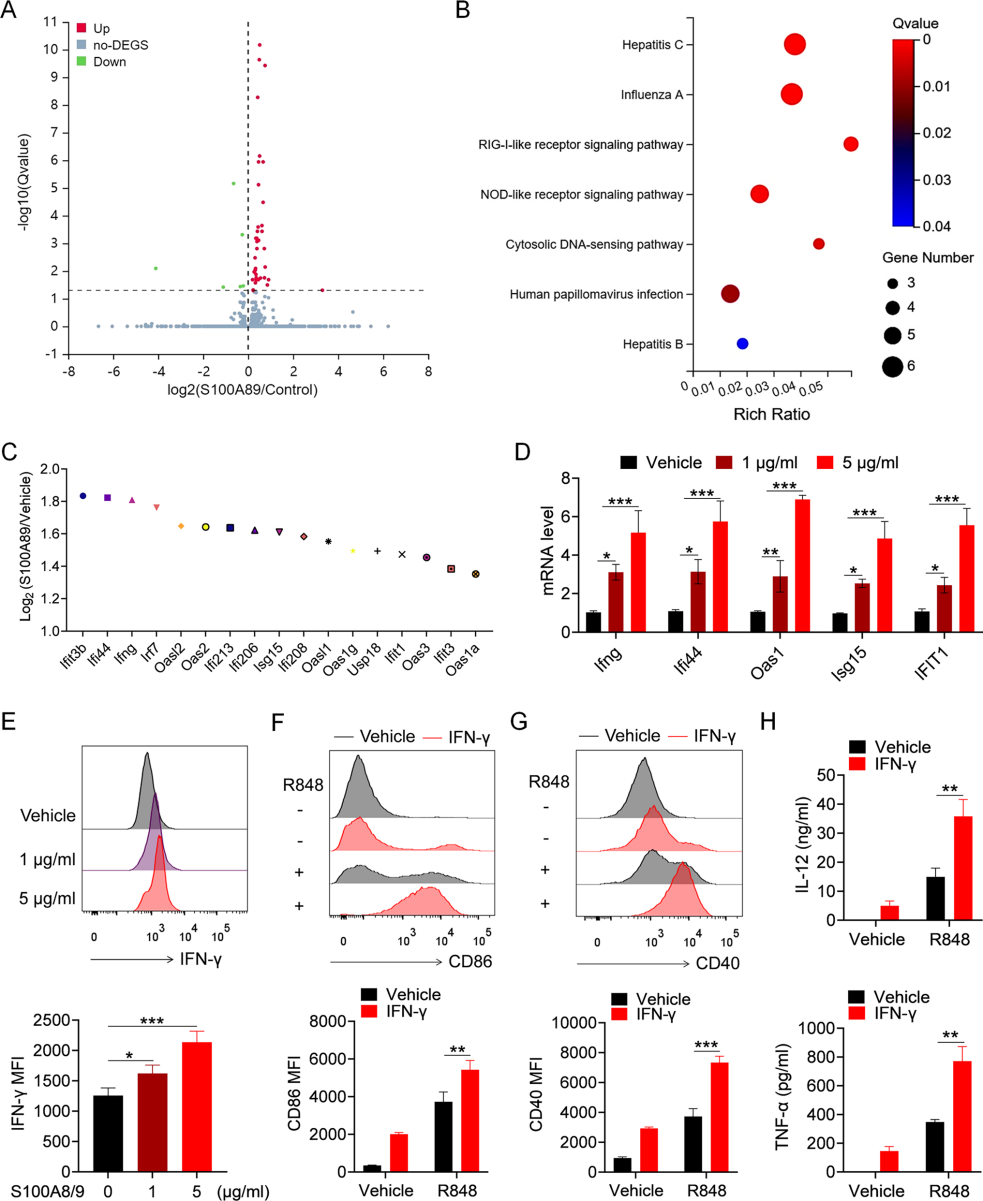
S100A8/9 augments the TLR7-mediated activation of BMDMs by promoting the secretion of IFN-γ. (A-C) BMDMs were treated with mouse recombinant S100A8/9 protein (1 μg/mL) or vehicle for 6 h, and then RNA-seq analysis was performed. **A** Differential expression genes at FDR < 0.05 in S100A8/9-treated BMDMs versus vehicle-treated BMDMs. **B** KEGG signaling pathway enrichment analysis of differential gene expression in S100A8/9-treated BMDMs versus vehicle-treated BMDMs. **C** The changes of the expression of IFN-γ and IFN-inducible genes in S100A8/9-treated BMDMs versus vehicle-treated BMDMs. **D**, **E** BMDMs were stimulated with mouse recombinant S100A8/9 protein (1 μg/mL and 5 μg/mL) for 6 or 12 h. The mRNA levels of IFN-γ and IFN-inducible genes were detected by Q-PCR (**D**). The protein level of IFN-γ was detected by FACS (**E**). **F–H** BMDMs were pretreated with mouse recombinant IFN-γ (20 ng/mL) for 12 h, followed by stimulation of R848 for 24 h. Flow cytometric analysis of the expression of CD86 and CD40 (**F**, **G**). ELISA analysis of the levels of IL-12/IL-23 p40 and TNF-α in culture supernatant (**H**). *p < 0.05, **p < 0.01, ***p < 0.001

To explore the mechanism by which S100A8/9 contributes to pathway activation in macrophages, we performed RNA-seq to analyze S100A8/9-induced gene expression in BMDMs (Fig. 5A). Notably, KEGG signaling pathway enrichment analysis revealed that the DEGs were associated mainly with IFN-related pathways (Fig. 5B). Among the top DEGs, the expression of IFN-γ and IFN-inducing genes in S100A8/9-treated BMDMs was significantly greater than that in vehicle-treated BMDMs (Fig. 5C). To further confirm the effect of S100A8/9 on IFN-γ secretion in BMDMs, mouse recombinant S100A8/9 protein was used to stimulate BMDMs, and the levels of IFN-γ and IFN-inducible genes were quantified. S100A8/9 significantly induced the expression of IFN-γ and IFN-inducible genes in BMDMs (Fig. 5D, E).

Next, we explored whether IFN-γ could promote TLR7 pathway activation. BMDMs were pretreated with mouse recombinant IFN-γ followed by stimulation with R848. IFN-γ significantly promoted R848-induced expression of CD86 and CD40 (Fig. 5F, G) and the secretion of IL-12 and TNF-α (Fig. 5H). These results suggest that S100A8/9 promotes TLR7 pathway activation in BMDMs by promoting the autosecretion of IFN-γ.

### S100A9 deficiency alleviates disease progression in IMQ-induced lupus mice

**Fig. 6:**
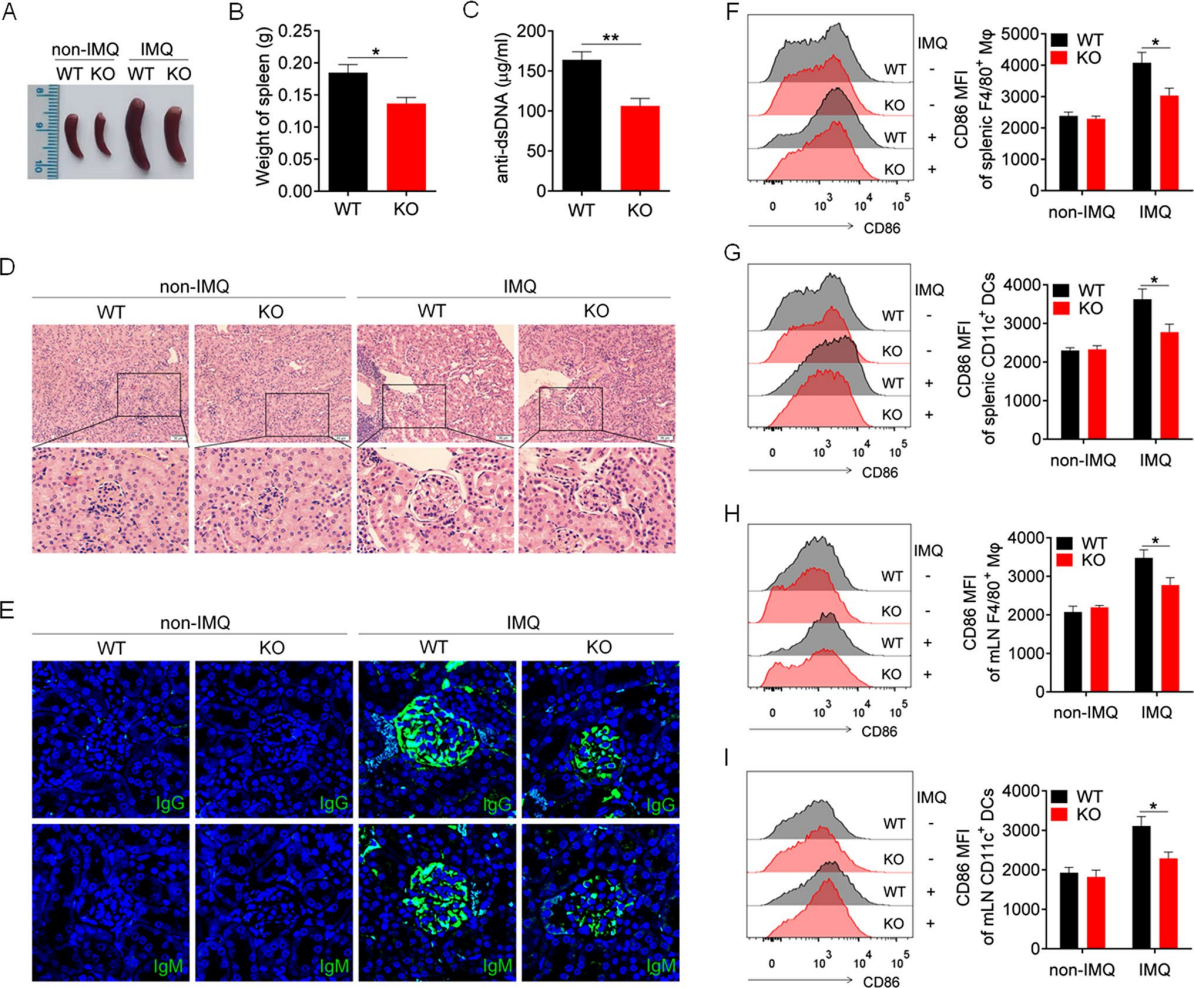
S100A9 deficiency alleviates IMQ-induced lupus in mice. S100A9.^−/−^ and wild-type mice (8-week-old) were treated with IMQ for 10 weeks, and killed for further experiment. **A** Representative images of marked splenomegaly. **B** Weight of spleens. **C** ELISA analysis of the anti-dsDNA antibody in serum. **D** Representative images of kidney sections detected by H&E. **E** Immunofluorescence of IgG and IgM of kidney sections. **F**–**I** Flow cytometric analysis of the expression of CD86 on macrophages and dendritic cells in spleens (**F**, **G**) and mLN (**H**, **I**) of mice. *p < 0.05

Previous studies have shown that targeted deletion of S100A9 results in the loss of S100A8 and S100A9 [31]. To further evaluate the ability of S100A8/9 to promote TLR7-mediated autoimmunity, S100A9-deficient mice (S100A9 KO mice) were generated, and in vivo studies were conducted. Compared with IMQ-treated wild-type mice, IMQ-treated S100A9 KO mice exhibited significantly reduced splenomegaly (Fig. 6A) and spleen weight (Fig. 6B). Enzyme-linked immunosorbent assay results revealed that the serum level of the anti-dsDNA antibody in IMQ-treated S100A9 KO mice was significantly lower than that in IMQ-treated wild-type mice (Fig. 6C). Kidney damage was significantly reduced in IMQ-treated S100A9 KO mice compared with IMQ-treated wild-type mice (Fig. 6D). We observed significantly reduced deposition of IgG and IgM antibodies in the subcutaneous and mesial regions of IMQ-treated S100A9 KO mice compared with IMQ-treated wild-type mice (Fig. 6E). These results suggest that S100A9 deficiency significantly alleviates disease progression in IMQ-induced lupus mice.

We further analyzed the effect of S100A9 deficiency on macrophage activation in IMQ-induced lupus mice. As expected, compared with IMQ-treated wild-type mice, IMQ-treated S100A9 KO mice presented significantly greater levels of CD86 and CD40 on splenic macrophages (Fig. 6F, Supplementary Fig. 5A) and DCs (Fig. 6G, Supplementary Fig. 5B). Moreover, S100A9 deletion significantly reduced the activation of splenic B cells (Supplementary Fig. 6A, B) and CD4^+^ T cells (Supplementary Fig. 6C, D) and reduced the percentage of germinal center B cells (Supplementary Fig. 6E, F) and plasmacytes (Supplementary Fig. 6G, H) in the spleen.

Similar results were also obtained in the lymph nodes, where S100A9 deletion significantly reversed the activation of macrophages (Fig. 6H, Supplementary Fig. 5C) and DCs (Fig. 6I, Supplementary Fig. 5D), reduced the activation of B cells (Supplementary Fig. 7A, B) and CD4^+^ T cells (Supplementary Fig. 7C, D) and decreased the percentage of germinal center B cells (Supplementary Fig. 7E, F) and plasmacytes (Supplementary Fig. 7G, H) in the mLNs of IMQ-induced lupus mice. These results indicate that S100A9 deficiency can reverse abnormal activation of the immune system and alleviate disease progression in mice with lupus induced by the TLR7 agonist IMQ.

### Lupus MDSC-derived S100A8/9 contributes to disease progression in IMQ-induced lupus mice

**Fig. 7:**
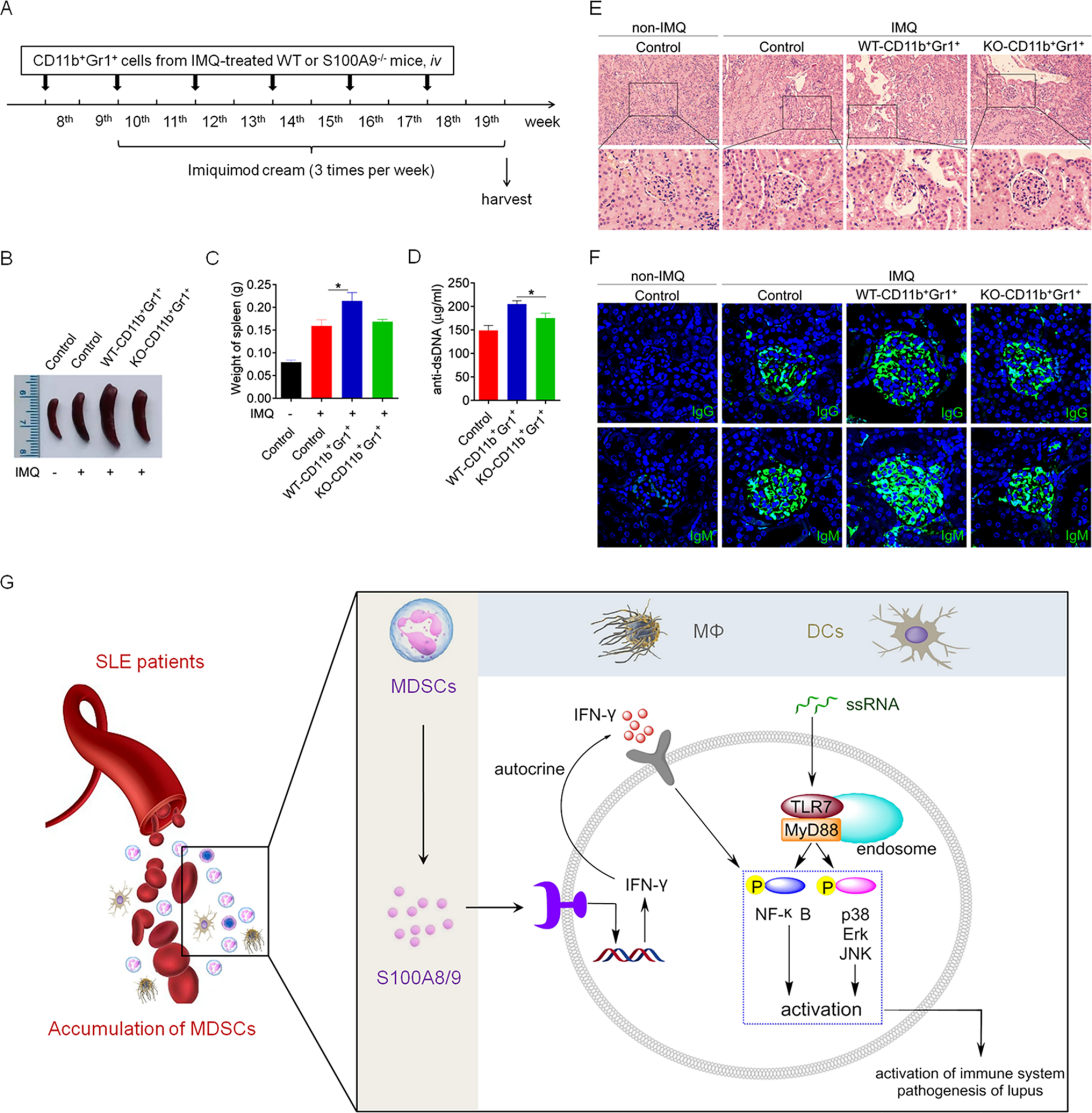
Lupus MDSCs-derived S100A8/9 contributes to the disease progression of IMQ-induced lupus mice. The splenic MDSCs (S100A9^−/−^-MDSCs and WT-MDSCs) were isolated from S100A9^−/−^ and wild-type mice which have been treated with IMQ for 10 weeks, and subsequently transferred into 8-week-old C57BL/6 mice by caudal vein (*n* = 6), 2 × 10^6^ cells/mice, once every 2 weeks. At the 10th week, mice were treated with IMQ to induce lupus model. After 10 weeks, the mice were killed for further study. **A** The schematic showing the experimental design. **B** Representative images of marked splenomegaly. **C** Weight of spleens. **D** ELISA analysis of the anti-dsDNA antibody in serum. **E** Representative images of kidney sections detected by H&E. **F** Immunofluorescence of IgG and IgM of kidney sections. *p < 0.05

To further investigate whether lupus MDSC-derived S100A8/9 contributes to TLR7-mediated autoimmunity, S100A9^−/−^ and wild-type mice were treated with IMQ for 10 weeks, after which splenic MDSCs (S100A9^−/−^-MDSCs and wild-type MDSCs) were isolated and adoptively transferred into IMQ-treated wild-type mice through the tail vein (Fig. 7A). As expected, compared with those of mice that received wild-type MDSCs, the mice that received S100A9^−/−^-MDSCs had markedly reduced splenomegaly (Fig. 7B), a decreased weight of the spleen (Fig. 7C), lower levels of anti-dsDNA antibody (Fig. 7D), a lesser amount of infiltration of lymphoid cells and diffuse expansion of the mesangial matrix in the kidneys (Fig. 7E), and reduced glomerular deposition of IgG and IgM antibodies (Fig. 7F).

Moreover, compared with those of mice that received wild-type MDSCs, the mice that received S100A9^−/−^-MDSCs showed markedly reduced activation of splenic B cells (Supplementary Fig. 8A, B) and CD4^+^ T cells (Supplementary Fig. 8C, D) and a decreased percentage of germinal center B cells (Supplementary Fig. 8E, F) and plasmacytes (Supplementary Fig. 8G, H) in the spleen. Similar results were also obtained coincidentally in the lymph nodes, where mice that received S100A9^−/−^-MDSCs showed markedly reduced activation of B cells (Supplementary Fig. 9A, B) and CD4^+^ T cells (Supplementary Fig. 9C, D) as well as decreased percentage of germinal center B cells (Supplementary Fig. 9E, F) and plasmacytes (Supplementary Fig. 9G, H) in the mLNs of IMQ-induced lupus mice. These results indicate that MDSC-derived S100A8/9 contributes to abnormal activation of the immune system and exacerbates disease progression in mice with lupus induced by the TLR7 agonist IMQ (Fig. 7G).

## Discussion

The role of MDSCs in the pathogenesis of autoimmune diseases has attracted increasing amounts of attention. However, their involvement in the regulation of TLR7-mediated autoimmune responses has not been previously reported. In this study, we confirmed that lupus MDSCs can promote TLR7-mediated activation of macrophages and DCs, thereby contributing to disease progression in mice with lupus induced by TLR7 agonist IMQ. Mechanistically, S100A8/9, secreted by lupus MDSCs, enhances IFN-γ production by BMDMs, subsequently promoting TLR7-mediated activation of macrophages and DCs. These findings offer molecular insights and suggest potential new treatment targets for SLE.

MDSCs, as immature myeloid cells, perform different immune functions under various pathological conditions, including tumor development, infection and autoimmune disease. Notably, our study and others have demonstrated that MDSCs play a pathogenic role in lupus pathogenesis: MDSCs increase abnormally in the early stage of lupus development, significantly earlier than those of Th17, Treg, T, and B cells, and can promote Th17 differentiation and other immune functions, indicating that MDSCs may be crucial immune cells involved in inducing lupus [23–25, 32]. Our previous study revealed that lupus MDSCs significantly promote IFN-I pathway activation in B cells [33], thus exacerbating the disease in lupus mice. Consistent with these findings, the present study showed that lupus MDSCs significantly worsened the disease in a lupus mouse model induced by the TLR7 agonist IMQ, further confirming that MDSCs play a critical pathogenic role in lupus pathogenesis and suggesting that targeting MDSCs may be an effective strategy for lupus treatment.

It has been proven that TLRs, especially TLR7 and TLR9, can indeed activate immune cells such as macrophages, DCs and B cells, and thereby affect the differentiation, activation, and function of immune cells. However, a number of studies in a mouse model of lupus have shown that TLR7 plays a pathogenic role in the pathogenesis of lupus, while TLR9 plays a protective role in the pathogenesis of lupus. Overexpression of TLR7 can induce systemic autoimmunity in normal mice, while deletion of TLR7 can significantly alleviate the pathogenesis of lupus in mice [12, 34]; TLR9 deletion significantly exacerbates the pathogenesis of lupus in mice [35, 36]. Specifically, TLR9 can restrict the function of TLR7, and the destruction of TLR9 function is conducive to TLR7 signaling and promotes the development of SLE [35]. Given that TLR7 plays an important role in the pathogenesis of lupus, we investigated the regulatory effects of lupus-derived MDSCs on the activation of TLR7 signaling. Another reason we focused on TLR7 signaling was that the IMQ-induced lupus model used in this study was mediated primarily by activation of the TLR7 pathway. Therefore, we studied the regulatory effect of lupus-derived MDSCs on the activation of TLR7 signaling.

Although the TLR7 pathway is well known for its key pathogenic role in SLE development, the regulatory mechanisms leading to abnormal activation of the TLR7 pathway have not been fully elucidated. The present study is the first to report that lupus MDSCs significantly promote the TLR7-mediated activation of macrophages and DCs, contributing to lupus pathogenesis. These findings help to elucidate the mechanism underlying the abnormal activation of the TLR7 pathway and open up new possibilities for the treatment of lupus and other autoimmune diseases. Additionally, TLR7 is involved in the occurrence and development of various other autoimmune diseases and immune-related conditions, such as rheumatoid arthritis and viral infections [37–39]. This study provides a theoretical basis for studying the pathogenesis of these diseases.

To investigate the role of MDSCs in lupus pathogenesis further, we performed RNA-seq to analyze the differences in gene expression in MDSCs from MRL/*lpr* mice at 6 weeks and 24 weeks of age. The results revealed 1130 genes with upregulated expression and 2225 genes with downregulated expression in 24-week diseased MRL/*lpr* mice. The 1130 genes with upregulated expression included proinflammatory factors such as S100A8/9, IL-6, and TNF-α, while the 2225 genes with downregulated expression included anti-inflammatory factors such as IL-10 and transforming growth factor-β, indicating that MDSCs in diseased MRL/*lpr* mice were in a proinflammatory state. Notably, previous studies reported that MDSCs in the spleens of NZBW F1 mice do not inhibit, but actually promote the proliferation and activation of CD4^+^ T cells [40]. These findings were consistent with the findings of the present study, suggesting that the immunosuppressive function of MDSCs in lupus is deficient.

As critical damage-associated molecular pattern proteins, S100A8/9 can interact with TLR4 and RAGE to trigger the intracellular NF-κB and MAPK signaling pathways [41, 42]. Although patients with SLE have higher serum and urine S100A8/9 levels than age-matched control subjects, the role of S100A8/9 in SLE pathogenesis has not been determined. Interestingly, one study reported that S100A9-deficient male NZBWF1 mice experienced more severe disease, while S100A9-deficient female NZBWF1 mice exhibited slightly less disease [30]. In the present study, S100A8/9 expression in MDSCs gradually increased as the age of the MRL/*lpr* mice increased. Moreover, S100A8/9 contributed to TLR7-mediated autoimmunity. However, this study did not determine the receptor to which S100A8/9 binds while promoting TLR7 pathway activation.

Furthermore, in this study, we investigated the mechanism by which S100A8/9 promotes TLR7-mediated abnormal activation of macrophages and DCs. The RNA-seq results suggested that S100A8/9 significantly promotes IFN-γ secretion from macrophages. IFN-γ, an important cytokine, can synergistically promote the activation of the TLR3 and TLR4 pathways and mediate immune cell activation [43, 44]. However, we did not further investigate the mechanism by which S100A9 promotes IFN-γ secretion in Mφ, but continued to investigate the effect of IFN-γ on TLR7-mediated activation of Mφ. S100A8/9 are known to possess proinflammatory functions consistent with those of the complex as a TLR4 ligand [45, 46]. Interestingly, several studies have shown that activation of TLR4 signaling can promote the secretion of IFN-γ in Mφ [47, 48]. Thus, we hypothesize that S100A8/9 may promote the secretion of IFN-γ from M*φ* through the TLR4 signaling pathway. However, additional in-depth studies are needed to confirm the role of S100A8/9 in regulating the secretion of IFN-γ in M*φ*.

In conclusion, this study demonstrated that lupus MDSCs promote TLR7 pathway activation, contributing to lupus pathogenesis through the S100A8/9-IFN-γ axis. These findings suggested that MDSC-derived S100A8/9 play important roles in TLR7-mediated autoimmunity. Our findings may offer valuable insights for SLE therapy by targeting MDSCs.

### Supplementary Information

Below is the link to the electronic supplementary material.

Supplementary file1 (PDF 2630 KB)

## Data Availability

Not applicable.

## Abbreviations

BMDCs: Bone marrow-derived dendritic cells
BMDMs: Bone marrow-derived macrophages
DCs: Dendritic cells
IFN-γ: Interferon gamma
IMQ: Imiquimod
MDSCs: Myeloid-derived suppressor cells
PBMCs: Peripheral blood mononuclear cells
S100A8: S100 calcium-binding protein A8
SLE: Systemic lupus erythematosus
TLRs: Toll-like receptors
