# Supplementary material for: MDSC-derived S100A8/9 contributes to lupus pathogenesis by promoting TLR7-mediated activation of macrophages and dendritic cells
Source: Cell Mol Life Sci. 2024 Mar 1;81(1):110. doi: 10.1007/s00018-024-05155-w (PMC10907481; doi:10.1007/s00018-024-05155-w)
Supplement: Supplementary file 1 — Supplementary file1 (PDF 2630 KB) [file 18_2024_5155_MOESM1_ESM.pdf]

Supplemental Data

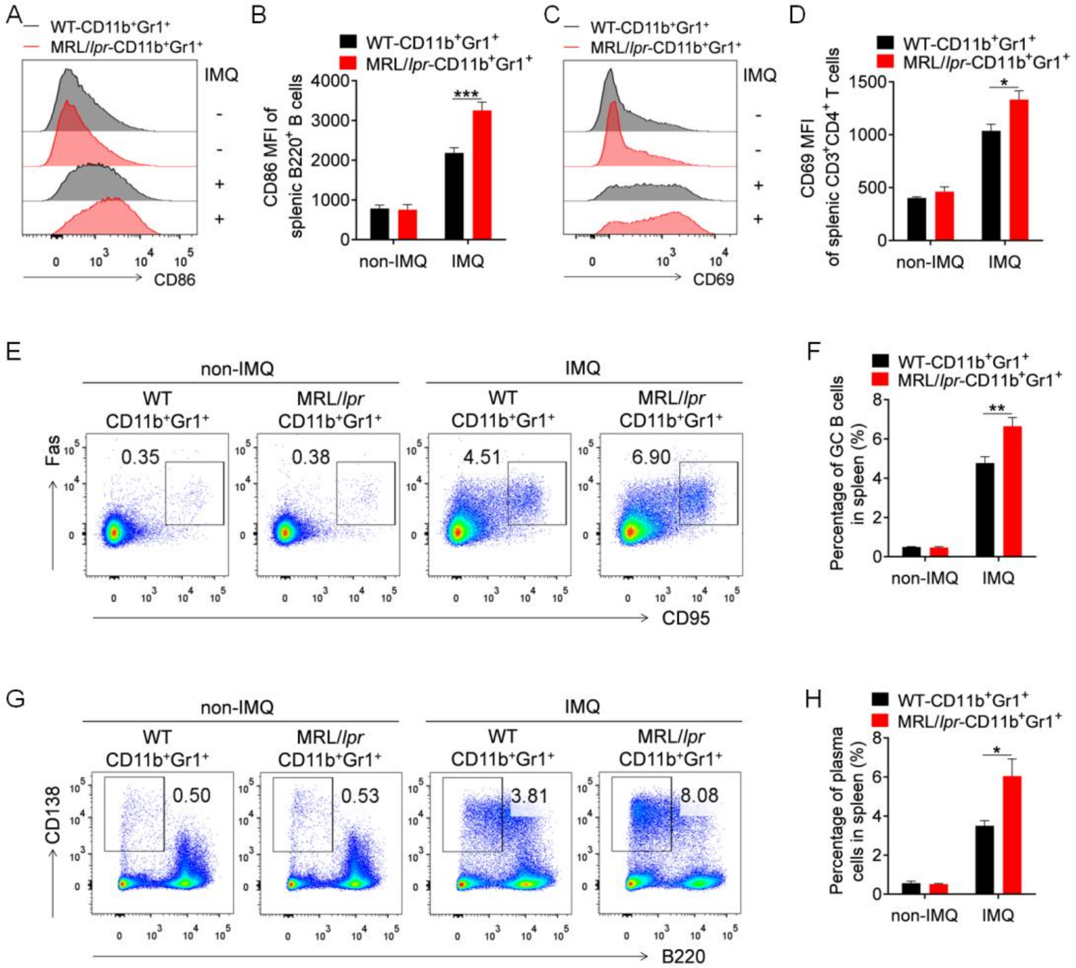

Supplementary Figure 1. Adoptively transfer of lupus MDSCs promotes the activation of immune cells in spleens of lupus mice. The splenic MDSCs isolated from 22-24 weeks old C57BL/6 mice or MRL/*lpr* mice were transferred into 8 weeks old C57BL/6 mice by caudal vein (n=6), 2×10^6 cells/mice, once every 2 weeks. At the 10th week, mice were treated with IMQ to induce lupus model. After 10 weeks, the mice were sacrificed for further study. **A, B** Flow cytometric analysis of the expression of CD86 on splenic B220$^+$ B cells. **C, D** Flow cytometric analysis of the expression of CD69 on splenic CD4$^+$ T cells. **E, F** Flow cytometric analysis of the percentage of germinal center B cells (B220$^+$GL7$^+$CD95$^+$) in splenic B220$^+$ B cells. **G, H** Flow cytometric analysis of the percentage of plasma cells (B220$^{-/low}$CD138$^+$) in splenocytes. * $p < 0.05$, ** $p < 0.01$, *** $p < 0.001$.

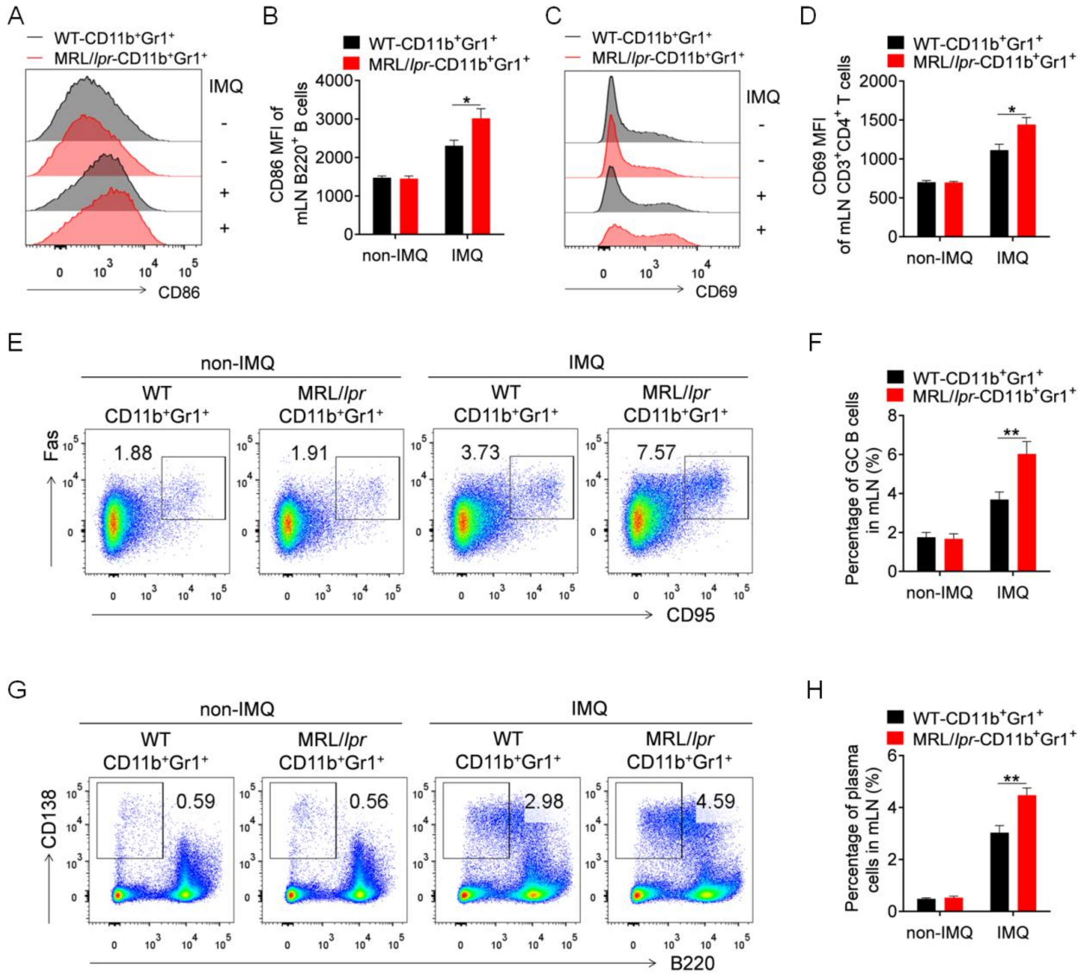

Supplementary Figure 2. Adoptively transfer of lupus MDSCs promotes the activation of immune cells in mLN of lupus mice. The splenic MDSCs isolated from 22-24 weeks old C57BL/6 mice or MRL/*lpr* mice were transferred into 8 weeks old C57BL/6 mice by caudal vein (n=6), 2×10^6 cells/mice, once every 2 weeks. At the 10th week, mice were treated with IMQ to induce lupus model. After 10 weeks, the mice were sacrificed for further study. **A, B** Flow cytometric analysis of the expression of CD86 on mLN $B220^+$ B cells. **C, D** Flow cytometric analysis of the expression of CD69 on mLN $CD4^+$ T cells. **E, F** Flow cytometric analysis of the percentage of germinal center B cells ($B220^+GL7^+CD95^+$) in mLN $B220^+$ B cells. **G, H** Flow cytometric analysis of the percentage of plasma cells ($B220^{-/low}CD138^+$) in mLN. * $p < 0.05$, ** $p < 0.01$, *** $p < 0.001$.

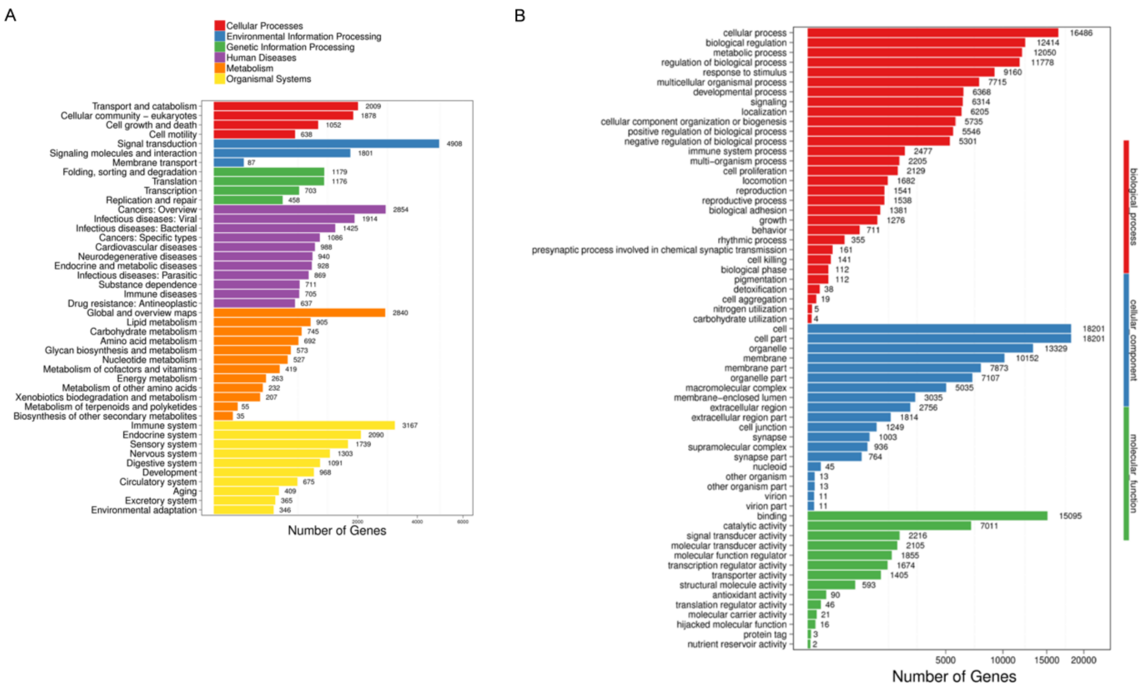

Supplementary Figure 3. Go analysis of up- and down-regulated DEGs. **A** GO analysis of represented biological pathways in the DEGs. **B** GO terms of Biological Process, Cellular Component, Molecular Function identifed by Panther GO-slim analysis.

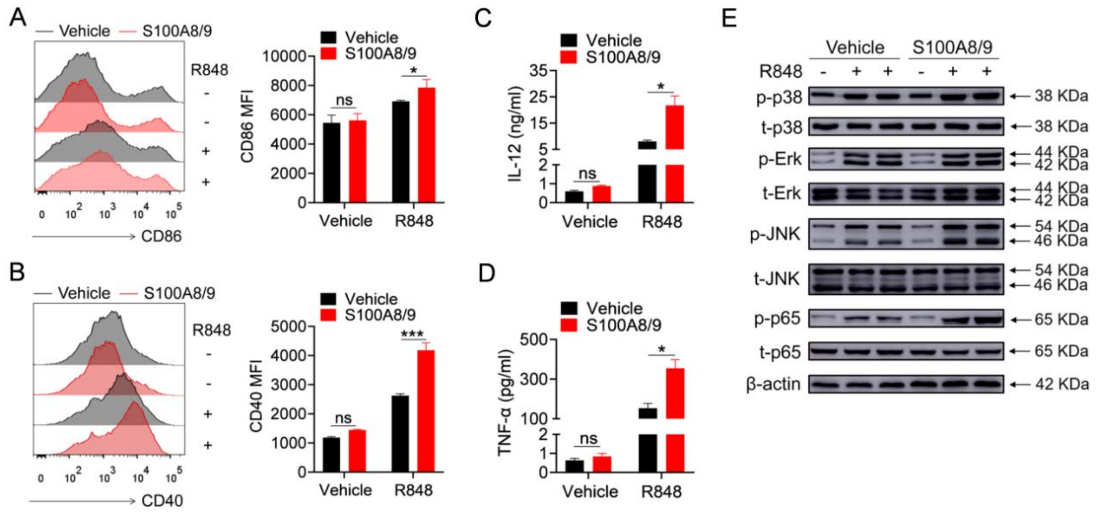

Supplementary Figure 4. S100A8/9 promotes TLR7-mediated BMDCs activation. BMDCs were pretreated with mouse recombinant S100A8/9 protein (1 μg/mL) for 12 h, and then stimulated with R848 (1 μg/mL). **A, B** Flow cytometric analysis of the expression of CD86 and CD40 at 24 h. **C, D** ELISA analysis of the levels of IL-12/IL-23 p40 and TNF-α in culture supernatant at 24 h. **E** Western blot analysis of the phosphorylation levels of p38, Erk, JNK and p65 at 30 and 60 min. * $p < 0.05$, *** $p < 0.001$.

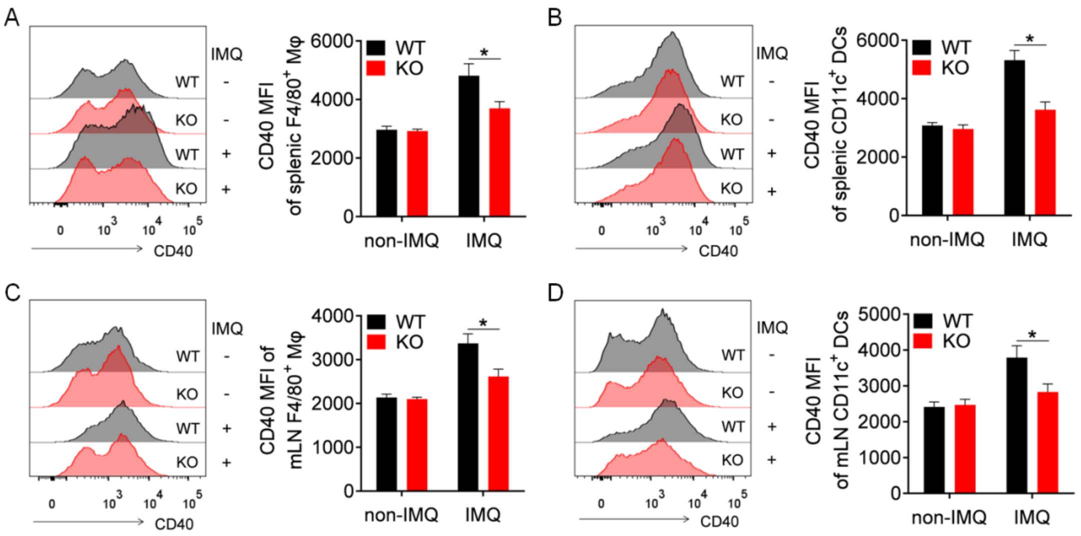

Supplementary Figure 5. S100A9 deficiency inhibits the expression of CD40 on macrophages and DCs in IMQ-induced lupus mice**.** S100A9$^{-/-}$ and WT mice at 8 weeks were treated with IMQ 3 times a week for 10 weeks, and sacrificed for further experiment. Flow cytometric analysis of the expression of CD40 on F4/80^{+} macrophages and CD11c^{+} dendritic cells in spleens (A, B) and mLN (C, D) of mice. * $p < 0.05$.

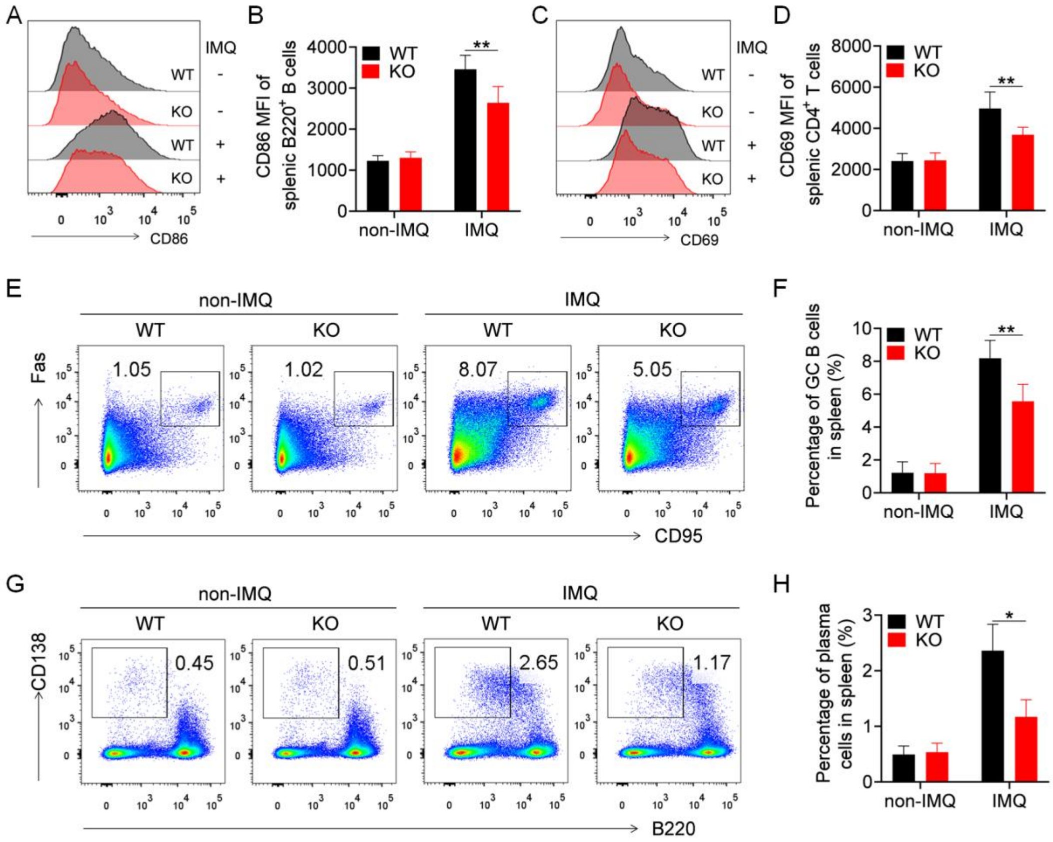

Supplementary Figure 6. S100A9 deficiency reduces activation of immune cells in spleens of lupus mice. S100A9$^{-/-}$ and wild-type mice (8 weeks old) were treated with IMQ for 10 weeks, and sacrificed for further experiment. **A, B** Flow cytometric analysis of the expression of CD86 on splenic B220^{+} B cells. **C, D** Flow cytometric analysis of the expression of CD69 on splenic CD4^{+} T cells. **E, F** Flow cytometric analysis of the percentage of germinal center B cells (B220^{+}GL7^{+}CD95^{+}) in splenic B220^{+} B cells. **G, H** Flow cytometric analysis of the percentage of plasma cells (B220$^{-/low}$CD138^{+}) in splenocytes. * $p < 0.05$, ** $p < 0.01$.

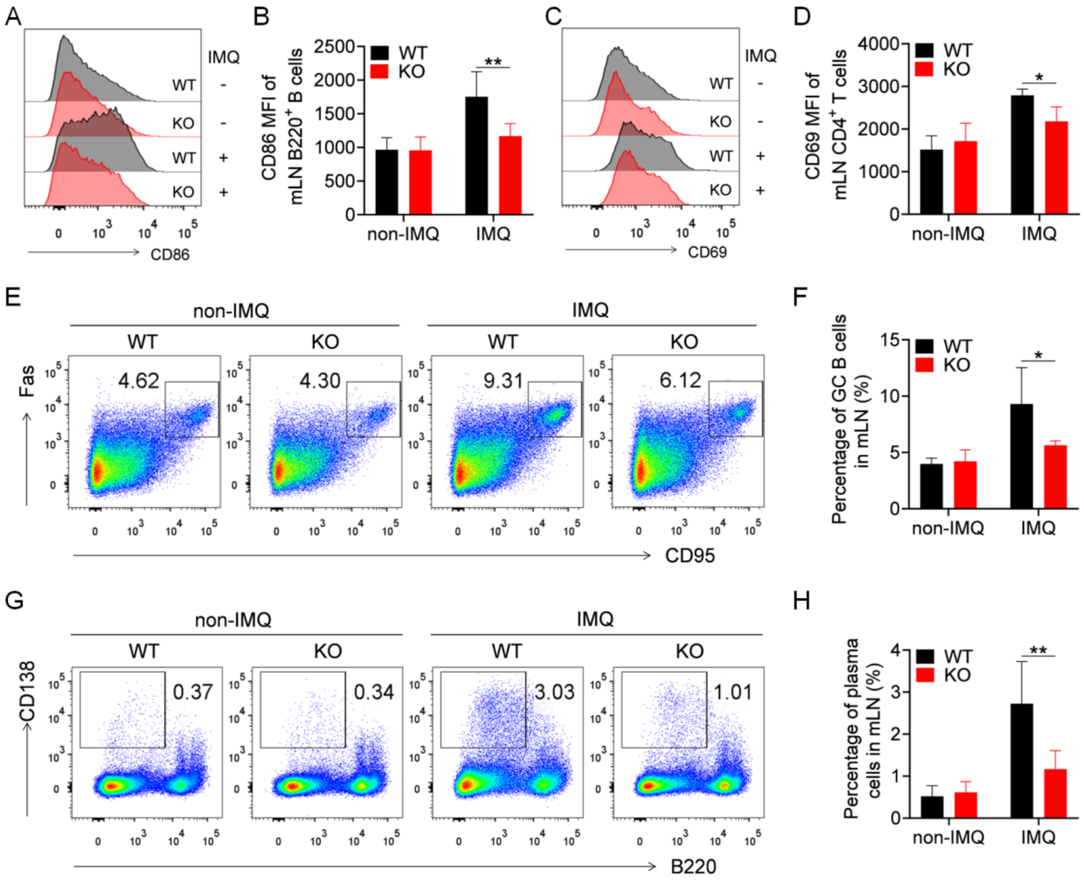

Supplementary Figure 7. S100A9-deficiency reduces activation of immune cells in mesenteric lymph nodes of lupus mice. S100A9$^{-/-}$ and wild-type mice (8 weeks old) were treated with IMQ for 10 weeks, and sacrificed for further experiment. **A, B** Flow cytometric analysis of the expression of CD86 on mLN B220^{+} B cells. **C, D** Flow cytometric analysis of the expression of CD69 on mLN CD4^{+} T cells. **E, F** Flow cytometric analysis of the percentage of germinal center B cells (B220^{+}GL7^{+}CD95^{+}) in mLN B220^{+} B cells. **G, H** Flow cytometric analysis of the percentage of plasma cells (B220$^{-/low}$CD138^{+}) in mLN. * $p < 0.05$, ** $p < 0.01$.

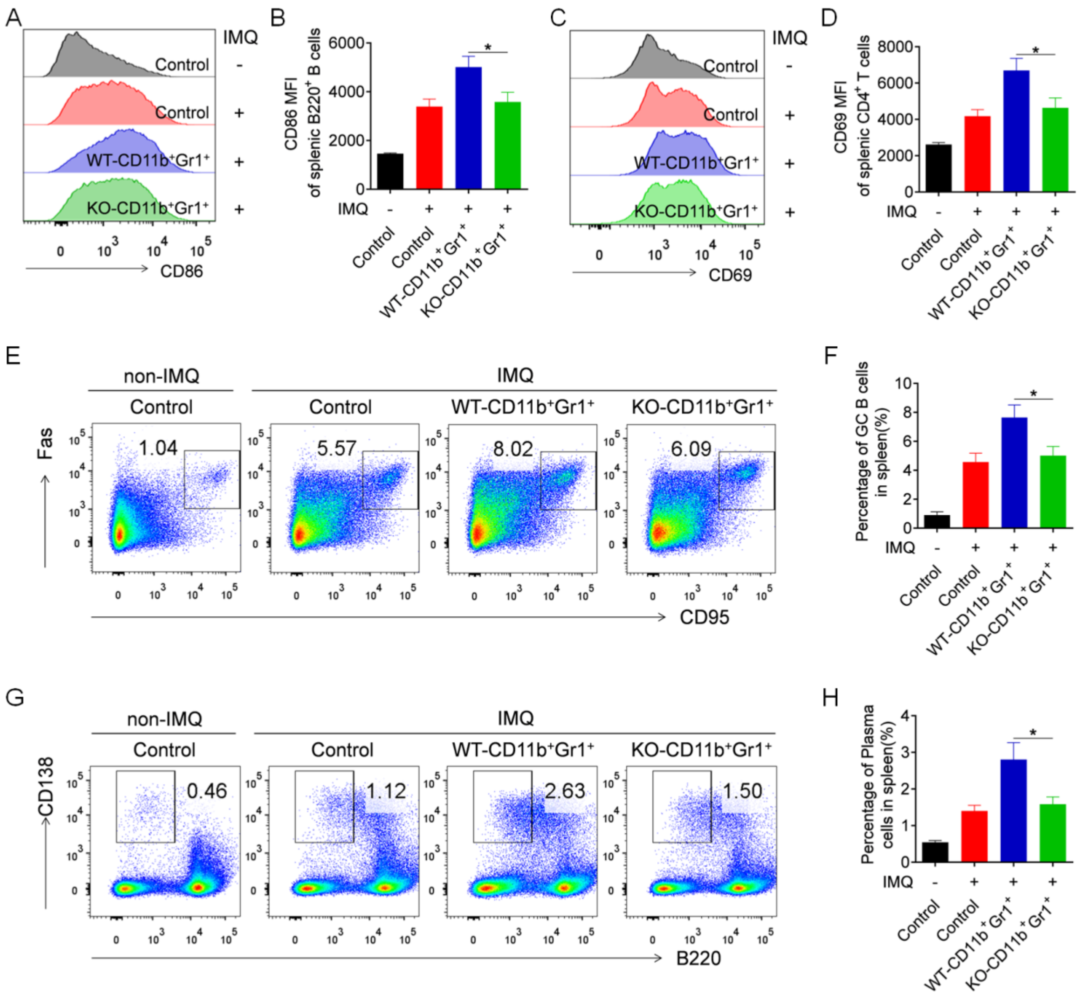

Supplementary Figure 8. Lupus MDSCs-derived S100A8/9 contributes to the activation of immune cells in spleens of lupus mice**.** The splenic MDSCs (S100A9$^{-/-}$-MDSCs and WT-MDSCs) were isolated from S100A9$^{-/-}$ and wild-type mice which have been treated with IMQ for 10 weeks, and subsequently transferred into 8 weeks old C57BL/6 mice by caudal vein (n=6), 2×10^{6} cells/mice, once every 2 weeks. At the 10th week, mice were treated with IMQ to induce lupus model. After 10 weeks, the mice were sacrificed for further study. **A, B** Flow cytometric analysis of the expression of CD86 on splenic B220^{+} B cells. **C, D** Flow cytometric analysis of the expression of CD69 on splenic CD4^{+} T cells. **E, F** Flow cytometric analysis of the percentage of germinal center B cells (B220^{+}GL7^{+}CD95^{+}) in splenic B220^{+} B cells. **G, H** Flow cytometric analysis of the percentage of plasma cells (B220$^{-/low}$CD138^{+}) in splenocytes. * $p < 0.05$.

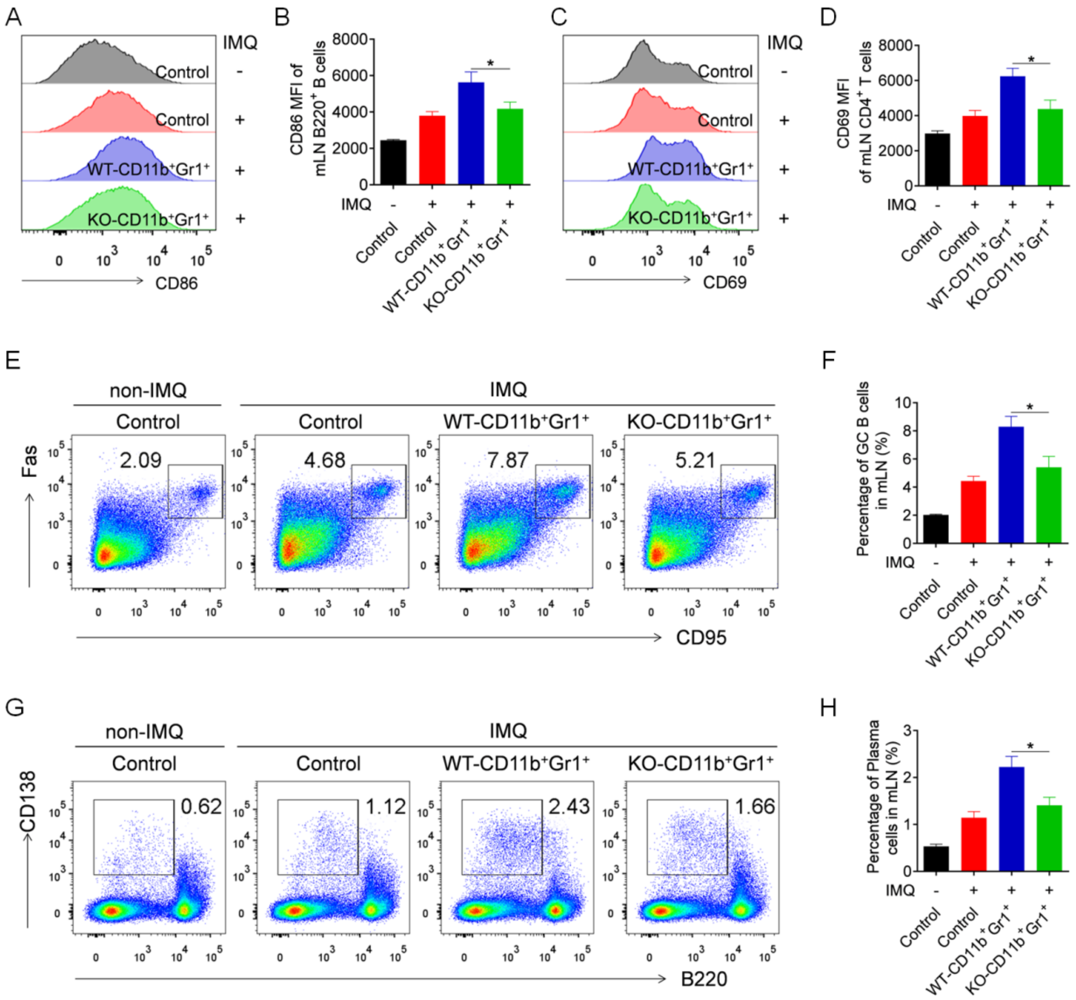

Supplementary Figure 9. Lupus MDSCs-derived S100A8/9 contributes to the activation of immune cells in mLN of lupus mice**.** The splenic MDSCs ($S100A9^{-/-}$-MDSCs and WT-MDSCs) were isolated from $S100A9^{-/-}$ and wild-type mice which have been treated with IMQ for 10 weeks, and subsequently transferred into 8 weeks old C57BL/6 mice by caudal vein (n=6), 2×10^6 cells/mice, once every 2 weeks. At the 10th week, mice were treated with IMQ to induce lupus model. After 10 weeks, the mice were sacrificed for further study. **A, B** Flow cytometric analysis of the expression of CD86 on mLN $B220^+$ B cells. **C, D** Flow cytometric analysis of the expression of CD69 on mLN $CD4^+$ T cells. **E, F** Flow cytometric analysis of the percentage of germinal center B cells ($B220^+GL7^+CD95^+$) in mLN $B220^+$ B cells. **G, H** Flow cytometric analysis of the percentage of plasma cells ($B220^{-/low}CD138^+$) in mLN. * $p < 0.05$.
